# A loss-of-function genetic screening reveals synergistic targeting of AKT/mTOR and WTN/β-catenin pathways for treatment of AML with high PRL-3 phosphatase

**DOI:** 10.1186/s13045-018-0581-9

**Published:** 2018-03-07

**Authors:** Jianbiao Zhou, Sabrina Hui-Min Toh, Zit-Liang Chan, Jessie Yiying Quah, Jing-Yuan Chooi, Tuan Zea Tan, Phyllis S. Y. Chong, Qi Zeng, Wee-Joo Chng

**Affiliations:** 1 Cancer Science Institute of Singapore, Singapore, Singapore; 2 Department of Medicine, Yong Loo Lin School of Medicine, National University of Singapore, Singapore, Singapore; 3 Translational Centre for Development and Research, National University Health System, Singapore, Singapore; 4 Institute of Molecular and Cell Biology, A*STAR (Agency for Science, Technology and Research), Singapore, Singapore; 5 Department of Hematology-Oncology, National University Cancer Institute of Singapore (NCIS), The National University Health System (NUHS), 1E, Kent Ridge Road, Singapore, 119228 Singapore

## Abstract

**Background:**

Protein tyrosine phosphatase of regenerating liver 3 (PRL-3) is overexpressed in a subset of AML patients with inferior prognosis, representing an attractive therapeutic target. However, due to relatively shallow pocket of the catalytic site of PRL-3, it is difficult to develop selective small molecule inhibitor.

**Methods:**

In this study, we performed whole-genome lentiviral shRNA library screening to discover synthetic lethal target to PRL-3 in AML. We used specific small molecule inhibitors to validate the synthetic lethality in human PRL-3 high vs PRL-3 low human AML cell lines and primary bone marrow cells from AML patients. AML mouse xenograft model was used to examine the in vivo synergism.

**Results:**

The list of genes depleted in TF1-hPRL3 cells was particularly enriched for members involved in WNT/β-catenin pathway and AKT/mTOR signaling. These findings prompted us to explore the impact of AKT/mTOR signaling inhibition in PRL-3 high AML cells in combination with WNT/β-catenin inhibitor. VS-5584, a novel, highly selective dual PI3K/mTOR inhibitor, and ICG-001, a WNT inhibitor, were used as a combination therapy. A synthetic lethal interaction between mTOR/AKT pathway inhibition and WNT/β-catenin was validated by a variety of cellular assays. Notably, we found that treatment with these two drugs significantly reduced leukemic burden and prolonged survival of mice transplanted with human PRL-3 high AML cells, but not with PRL-3 low AML cells.

**Conclusions:**

In summary, our results support the existence of cooperative signaling networks between AKT/mTOR and WNT/β-catenin pathways in PRL-3 high AML cells. Simultaneous inhibition of these two pathways could achieve robust clinical efficacy for this subtype of AML patient with high PRL-3 expression and warrant further clinical investigation.

**Electronic supplementary material:**

The online version of this article (10.1186/s13045-018-0581-9) contains supplementary material, which is available to authorized users.

## Background

Acute myeloid leukemia (AML) is a group of heterogeneous diseases, arising from clonal expansion of transformed hematopoietic stem and progenitor cells [1]. Depending on the subtypes of leukemia and the underlying genetic defects, the 5-year overall survival rate ranges from 30 to 40% for de novo AML [2]. The current standard treatment is still chemotherapy, used over the last decades [3, 4].

Signaling transduction initiated by protein phosphorylation and dephosphorylation governs a host of fundamental cell functions, such as proliferation, growth, survival, and apoptosis [5, 6]. This phospho-switch process is mediated by kinases and phosphatases, respectively. Phosphatase of regenerating liver 3 (PRL-3) (encoded by protein tyrosine phosphatase type IVA 3, PTP4A3) is one member of VH1-like protein tyrosine phosphatase (PTP) with dual specificity family [7–9]. A growing body of evidence indicates that aberrant expression of PRL-3 plays an essential role in the process of cancer development and progression [10–13]. Our group first reported that PRL-3 protein is overexpressed in about half of bone marrow samples of AML patients, while its expression is negative in normal myeloid cells [14]. Notably, elevated expression of PRL-3 is associated with poor survival of AML patients [15–18]. Thus, PRL-3 represents an attractive target for treating AML [18, 19].

However, specific PRL-3 inhibitor is not available in any advanced stage of drug development pipeline [19]. The active pocket of PRL-3 is shallow and hydrophobic, which makes it difficult for small molecule inhibitors to be fully incorporated [20, 21]. Furthermore, the amino acid sequence similarity of PRL-3 compared with the other two PRL family members, PRL-1 and PRL-2, is high, so the specificity of these inhibitors is an issue [22]. These two reasons above limit the success of PRL-3-specific inhibitors in clinical development. Thus, an indirect approach should be taken to target PRL-3 for cancer treatment. It is known that the activity of phosphatases is often under control of other protein regulators, and certain pathways or oncogenes are specifically activated by cancer-associated phosphatases. PRL-3-positive cancer cells may be especially reliant on these regulators or pathways to sustain their oncogenic properties. Therefore, these dependencies offer the possibility of targeting PRL-3 via manipulation of these upstream regulators or addicted pathways.

In this study, we performed a pooled, whole-genome shRNA library in one pair of isogeneic AML cell lines, TF1-pEGFP (vector control), and TF1-hPRL3 (overexpression). We identified a synthetic lethal interaction between inhibition of AKT/mTOR and WNT/β-catenin pathways and validated the synergism of the co-target treatment on the growth inhibition of AML cells in vitro and in vivo.

## Methods

### Cell lines and cell culture

The isogenic cell line TF1-pEGFP and TF1-hPRL3 have been characterized and reported previously [23]. AML cell lines, including MOLM-14 and OCI-AML2, were grown in RPMI1640 (Invitrogen, Carlsbad, CA) supplemented with10% of fetal bovine serum (FBS, JRH Bioscience Inc., Lenexa, KS) at density of 2 to 10 × 10^5^ cells/ml in a humid incubator with 5% CO_2_ at 37 °C. Bone marrow blast cells (> 90%) from newly diagnosed AML patients were obtained at National University Hospital in Singapore with informed consent. This study was approved by Institutional Review Board (IRB) of National University of Singapore. Primary AML cells were cultured in IMDM with 10% FBS, FLT3 ligand (20 ng/ml), SCF (20 ng/ml), IL-3 (20 ng/ml), G-CSF (50 ng/ml), TPO (50 ng/ml), and 1% penicillin/streptomycin. Human cytokines were purchased from Peprotech (Rocky Hill, NJ).

### PRL-3 gene expression and survival analysis

PTP4A3 gene expression data was extracted from RNA-seq FPKM value of TCGA cohort. The associated clinical data was also downloaded from the Firehose of Broad GDAC (http://gdac.broadinstitute.org/) version 2016_01_28. Kaplan-Meier analysis and log-rank test were computed using GraphPad Prism^®^ version 5.04 (GraphPad Software; La Jolla, CA). In these survival analyses, cases were stratified into high (≥ median) and low (< median) expression groups, or first quantile (Q1, lowest 25%) and fourth quantile (Q4, highest 25%) expression groups.

### Pooled short-hairpin RNA library screen

TF1-pEGFP and TF1-hPRL3 cells were transduced in replicates with a pooled shRNA library (MISSION^®^ shRNA), consisting of 80,000 hairpins targeting 16,000 genes at an MOI of 0.3 hairpins/cell or negative control scramble shRNA. After 48 h, puromycin was added to the culture media at concentration of 5 μg/ml to select for transduced cells. Puromycin was continuously added to the media until cells were harvested at day 12. Genomic DNA was isolated by using DNeasy Blood & Tissue Kit (Qiagen,Germany) from 5 million cells of each pool and scramble shRNA-transduced cells. Each 100 ng of DNA per pool was used as input for PCR amplification of shRNA sequences by LAP1 amplification primer: 5′-TACAAAATACGTGACGTAGAAA3′ and LAP2 amplification primer: 5′-TTTGTTTTTGTAATTCTTTA-3′. The purified PCR products were used as DNA templates. Sequencing libraries were created using Rubicon ThruPLEX® DNA-seq Kit (R400406) following the manufacturer instruction. Briefly, 44 input DNA samples were end repaired and stem-loop adaptors are ligated to the 5′ and 3′ ends of the DNA fragments. High-fidelity PCR was performed to incorporate Illumina-compatible indexes to each DNA library. The constructed libraries were quantified using Agilent DNA High Sensitivity Chip (5067-4626) on Agilent Bioanalyser (G2940CA). Sequencing was performed by Macrogen Inc. (60-24 Gasan-dong Geumchun-gu, Seoul Korea) on a MiSeq using 300-bp paired-end processing.

### Data processing

Replicates of cell lines transfected with pooled library and negative control shScramble were subjected to shRNA-seq using Agilent 300-bp pair-end library. The quality of the sequences was checked using fastqc v0.11.1. Upon quality check, the sequences in the fastq files were searched for perfect matching to the TRC library database dated April 5, 2011, from RNAi consortium website. The mapping percentage ranges from 11 to 17% for the replicates and 0.12% for the negative control shScramble. Unmapped sequences were removed from further analyses. Correlation *Rho* among the replicates of pEGFP and hPRL3 is in the range of + 0.17 to + 0.19 as computed by Spearman correlation coefficient test. The counts from the mapping analysis were then extracted. Hairpins with less than 10 counts across the samples were filtered out. The counts data were then normalized to variance-stabilizing transform using the R 3.1, Bioconductor package Deseq v1.22.1. Subsequently, to get differentially expressed hairpins and genes, RIGER extensions in the GENE-E v3.0.204 package was employed, using default parameter settings. *p* value < 0.05 is deemed significant.

### Small molecule inhibitors

AKT/mTOR dual inhibitor, VS-5584, was synthesized by S*Bio (Singapore). Wnt/β-catenin inhibitor, ICG-001, was purchased from ApexBio (Houston, TX). All the inhibitors were dissolved in dimethyl sulfoxide (DMSO) to make 10 mM stock and keep in − 20 °C before use.

### Cell viability assays

AML cell lines or primary AML cells were seeded in 96-well culture plates at a density of 2 × 10^4^ viable cells/100 μl/well in triplicates. CellTiter-Glo® Luminescent Cell Viability Assay (CTG assay, Promega, Madison, WI) was used to determine the cell growth and viability as previously described [14]. Each experiment was in triplicate.

### Real-time quantitative reverse transcriptase-PCR (qRT-PCR)

To quantify gene expression, RNA samples were extracted from relevant AML cell lines. Typically, 1 μg of total RNA was used to generate cDNA by using SuperScript^®^ III RT (Thermo Fisher Scientific) with oligo-dT primer. qRT-PCR was performed using the Power SYBR^®^ Green PCR Master Mix as recommended by the manufacturer (Applied Biosystems). GAPDH was used as the internal control. SDS 2.2.1 software (Applied Biosystems) was used to perform relative quantification of target genes using the comparative C_T_ (ΔΔC_T_) method. The primer sequences for PRL-3 gene are PRL-3-Forward: 5′-AGAAGGATGGCATCACCGTTGT-3′ and PRL-3-Reverse: 5′-ACTTCACGC TCTCAATAAGCG-3′.

### Combination index and isobologram analysis

The calculation of combination index (CI) and isobolograms with the CalcuSyn software was reported in our publication before [24]. Briefly, the CI values were calculated according to the levels of growth inhibition (fraction affected, Fa) by each agent individually and combination of VS-5584 with ICG-001. Isobolograms, which indicate the equipotent combinations of different dose (ED_50_, ED_75_, and ED_90_, etc.), were used to illustrate synergism (CI < 1), antagonism (CI > 1), and additivity (CI = 1). Constant ratio combinations of the two drugs at 0.25×, 0.5×, 1×, 2×, and 4× of their ED_50_ was used. Three independent studies were conducted for each combination.

### Apoptosis assays

Two million cells were stained with annexin V-FITC and propidium iodide (PI) according to the manufacturer’s instruction (BD Pharmingen, San Diego, CA) and analyzed using a BD LSR2 flow cytometer (Franklin Lakes, NJ) and the Flowjo software (Tree Star, Inc., Ashland, OR).

### Western blot analysis

AML cell lines were treated with different small molecule inhibitors for 48 h. Cells were harvested and lysed with radio-immunoprecipitation assay (RIPA) buffer (20 mM HEPES at pH 7.4, 1% Triton X-100,150 mM NaCl, 1 mM EDTA, 1 mM EGTA, and 1X Protease Arrest). Total protein concentrations were determined using the Bio-Rad protein assay (Bio-Rad Laboratories, Inc., Hercules, CA) and SDS-PAGE and immunoblot analyses performed with different antibodies. Antibodies used were as follows: anti-AKT, phospho-AKT (Ser427), Survivin, full length and cleaved poly (ADP-ribose) polymerase (PARP), caspase 3, and caspase 7 from Cell Signaling Technologies, Inc. (Danvers, MA) and anti-Actin from Santa Cruz Biotechnology (Santa Cruz, CA).

### Evaluation of combination therapy in leukemia-engrafted mice

Six- to 8-week-old female NOD/SCID mice were purchased from In Vivos (Singapore) and maintained in specific pathogen-free conditions. As a standard procedure to improve the engraftment efficiency, mice were given Endoxan® (Cyclophosphamide, Baxter Oncology GmbH, Germany) 150 mg/kg/day for 2 days followed by one rest day before leukemia cells were injected [25]. MOLM-14 cells (3 × 10^6^) or OCI-AML2 cells (3 × 10^6^) were injected into the tail vein of the mice. Mice were treated with either vehicle control or VS-5584 (5 mg/kg/day) or ICG-001 (50 mg/kg/day) or VS-5584 and ICG—01 in combination by intraperitoneal (I.P.) in 100 μL volume from the second day. Ten mice were in each group.

At the end of survival experiments, bone marrow cells were harvested and proceeded to FACS analysis of human CD45 marker. The protocol was reviewed and approved by Institutional Animal Care and Use Committee in compliance to the guidelines on the care and use of animals for scientific purpose.

### Statistical analysis

The experimental results presented in the figures in the “Results” section were representative of duplicate or triplicate observations. The data were presented as the mean values ± standard derivation of the mean (SD). Comparisons between two groups were evaluated by *t* test. Multiple comparisons of more than two groups were evaluated by two-way analysis of variance (ANOVA), and *p* values of < 0.05 were considered to be significant. Survival analysis was performed by Kaplan-Meier analysis (SPSS, ver.12). Survival curves of the different treatment groups were compared using the log-rank test (*p* < 0.05).

## Results

### High expression of PRL-3 is an independent prognostic factor in AML

**Fig. 1:**
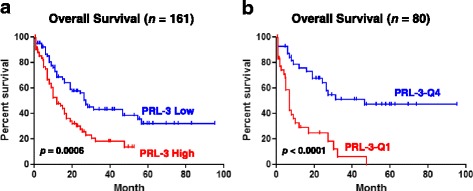
PRL-3 expression level confers prognostic value for AML patients. **a** Overall survival analysis of patients with PRL-3 high vs PRL-3 low. **b** Overall survival analysis of patients with PRL-3 Q4 vs PRL-3 Q1. Survival curves were constructed according to the Kaplan-Meier method. PRL-3 high ≥ median value; PRL-3 low < median value. Statistical significance was considered when *p* < 0.05. HR hazard ratio

We and others reported that PRL-3 is an independent prognostic factor in AML based on microarray data of human AML [15–18]. Recently, RNA-seq technology emerges as superior whole-genome transcription profiling method, which delivers low background signal, and enables a more sensitive and accurate quantification of expression levels than microarray platform. Here, we examine the prognostic ability of PRL-3 in publicly available RNA-seq data of human AML. TCGA RNA-seq and clinical data were downloaded from Firehose of Broad GDAC (http://gdac.broadinstitute.org/) version 2016_01_28. Total 166 AML patients had survival data. If all AML patients were divided into two groups according to the median expression level of PRL-3 (PRL-3 low: < median level; PRL-3 high: > median level), a significant difference was observed in overall survival between these two groups (log-rank *p* = 0.0006). The median survival period for PRL-3 high group was 12.2 vs 27.37 months for PRL-3 low group (HR = 2.13, 95% CI 1.38–2.28) (Fig. 1a). If patients were divided into four groups in terms of PRL-3 expression, we observed more pronounced difference in the overall survival in group with highest PRL-3 expression (PRL-3-Q4) vs group with lowest expression (PRL-3-Q1) (log-rank *p* = 0.0001). The median survival period for PRL-3-Q4 group was 7.1 vs 46.7 months for PRL-3-Q1 group (HR = 4.54, 95% CI 2.36–8.73) (Fig. 1b). In a multivariate analysis that considered clinically important factors which were present at diagnosis, such as age, CD34 positivity, leukocyte count, complex karyotype (cytogenetics), and FAB (French-American-British) subtype, PRL-3 expression level remained statistically significant and was shown to be independently associated with worse overall survival (*p* = 0.043). In conclusion, high expression of PRL-3 is associated with a shorten survival length, suggesting an independent, poor prognostic factor for AML patients.

### Genome-wide shRNA synthetic lethal screen against the PRL-3 oncogene

**Fig. 2:**
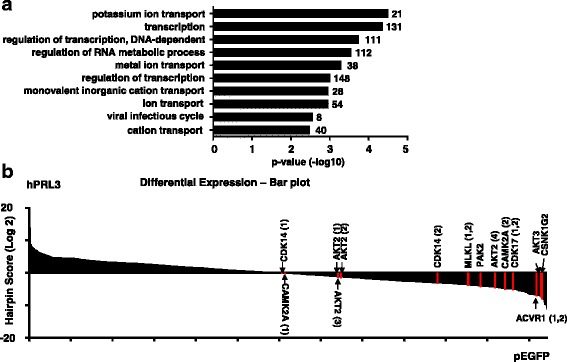
Functional diversity of PRL-3 synthetic lethal genes. **a** Bar chart showing the enriched functional classification of candidate genes (*y*-axis) in Additional file 1: Table S1 based on biological processes as annotated in Gene Ontology Consortium (www.geneontology.org) and the corresponding *p* value in –log10 scale (*x*-axis). The *p* value denotes selective enrichment for genes in the corresponding biological process. The number of genes was given next to the bar. **b** Pooled negative-selection screening in hPRL3 overexpressing leukemia cell line, depicting changes in representation of informative shRNAs (*x*-axis) during 12 days of culture. The hairpins are aligned from the highest to lowest hairpin score (*y*-axis). Highlighted shRNAs in bar plot are protein kinases which are critical for the survival of PRL-3 overexpressing leukemia cells. The highlighted shRNAs are based on RIGER analysis results and not all shRNAs targeting a gene are reported

We utilized a pooled lentiviral shRNA library for screening oncogenes or pathways are essential for the survival of AML cells overexpressing PRL-3 phosphatase in the isogenic pair of TF1-pEGFP and TF1-hPRL3 cell lines (Additional file 1: Table S1). We analyzed the change in relative abundance of each shRNA between TF1-pEGFP and TF1-hPRL3 cell after they were normalized with scramble shRNA controls, respectively. Using criteria of *p* value < 0.05, a total of 819 genes were found to be essential genes in TF1-hPRL3 cells, as shown by the significant depletion in abundance of the hairpins targeting these genes. The full list of these 891 genes and hairpin IDs were included in Additional file 1: Table S1. The list of these candidate genes was functionally diverse. We also performed gene ontology (GO) enrichment analysis and found several biological processes, including “transcription”, “transcription regulation”, and “transcription regulator activity”, which were significantly related to this gene list (*p* = 2.0E−6, 3.1E−06, and 5.8E−06, respectively) (Fig. 2a).

As protein kinases represent one of the most successful class of drug target in the field of cancer, we identified eight protein kinases in our list, including CSNK1G2 (casein kinase 1 gamma 2), ACVR1 (activin A receptor type 1), PAK2, and AKT3 (V-Akt murine thymoma viral oncogene homolog 3), AKT2, CAMK2A (calcium/calmodulin-dependent protein kinase II alpha), MLKL (mixed lineage kinase domain-like), CDK14 (cyclin-dependent kinase 14), and CDK17 (Fig. 2b). This gene list was particularly enriched for members involved in WNT/β-catenin pathway signaling, including FRAT2 (GSK-3 binding protein FRAT2), TMED3 (transmembrane P24 trafficking protein 3), WNT9A, WNT9B, WNT10B, TCF7L2 (transcription factor 7 like 2), and SFRP1 (secreted frizzled-related protein 1) (Additional file 1: Table S1).

### AKT and WNT/β-catenin pathways are key for the survival of AML cells with PRL-3 overexpression

It has been reported that PRL-3 activation triggers epithelial-mesenchymal transition (EMT) and resistance to stress-induced apoptosis through increasing PI3K/AKT function [26–28]. Furthermore, recent findings demonstrate that PRL-3 increases mTOR translocation to lysosomes via increased mTOR binding affinity to Rag GTPases, thus directly activating mTORC1, which is concomitantly a downstream effector of PI3K/AKT signaling pathway. The WNT/β-catenin pathway has been shown to play an essential role in the normal hematopoiesis and deregulation of this pathway resulted in the transformation of leukemia stem cells (LSCs) in AML [29, 30]. Taken together, these data suggest that PI3K/ATK/mTOR and WNT/β-catenin pathways could be required for the development of PRL-3 high AML.

**Fig. 3:**
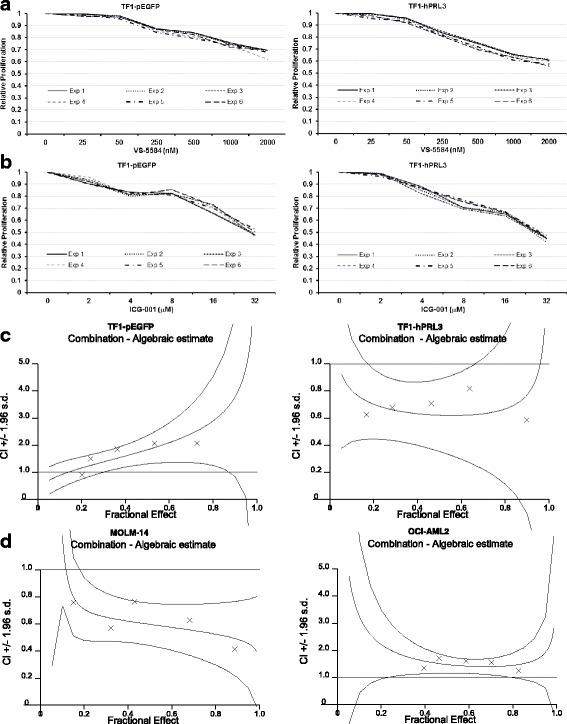
Cell proliferation assay analysis of VS-5584, ICG-001 monotherapy, and combination treatments on AML cell lines. TF1-pEGFP and TF1-hPRL3 cells were incubated with either DMSO control or increasing concentration of VS-5584 (**a**) and ICG-001 (**b**) for 48 h, followed by CTG assays. The treated results were shown in percentage after normalization with their DMSO control (100%), respectively (*n* = 6, mean ± SD). **c** TF1-pEGFP and TF1-hPRL3 cells and **d** MOLM-14 and OCI-AML2 cells were treated for 48 h with either single agent (VS-5584 or ICG-001 alone) or in combination at the indicated concentrations. Cell viability was then analyzed by CTG assays (*n* = 3, mean ± SD). These isobolograms of combination index (CI) for combination of VS-5584 and ICG-001 were generated by CalcuSyn software. A straight dot line represents an additive affect, where CI = 1. Any CI value which falls below this line is less than 1, indicating synergistic effect from the two drug combination. Antagonism is implied by CI > 1, where points are located above the dot line

These abovementioned findings, together with our gene list enriched with WNT-related molecules and AKT2, AKT3 genes, prompted us to explore the impact of PI3K/AKT/mTOR signaling inhibition in PRL-3 high AML cells in combination with WNT/β-catenin inhibitor. VS-5584 is a novel, highly selective dual PI3K/mTOR inhibitor and has obtained the Food and Drug Administration (FDA, USA) approval as orphan drug for mesothelioma patients [31]. The small molecule ICG-001 competes with β-catenin for binding CREB-binding protein (CBP), thus inhibiting CBP function as a co-activator of Wnt/β-catenin-mediated transcription [32]. ICG-001 is currently being evaluated in early phase clinical trial for solid tumors and leukemia. We took advantage of the selectivity and potency of these two inhibitors to investigate the combination therapy of these two inhibitors in PRL-3 high AML cells. To determine the effects of combination treatment of the AKT/mTOR inhibitor VS-5584 and the WNT inhibitor ICG-001 on human AML cell proliferation, we first treated TF1-pEGFP and TF1-hPRL3 cells with various concentrations of VS-5584 and ICG-001 as single drug and in combination and monitored cell proliferation by CTG assay. Figure 3a, b showed that VS-5584 or ICG-001 alone induced a dose-dependent inhibition of proliferation with IC_50_ values of 2.4 and 22 μM respectively, on TF1-pEGFP cells, as well as 2 and 18 μM on TF1-hPRL3 cells. To examine the synergism of these two drugs, we treated TF1-pEGFP and TF1-hPRL3 cells with a combination of VS-5584 and ICG-001 in a constant ratio to one another and generated Fa-CI plots using CalcuSyn software. As shown in Fig. 3c, all the experimental points had CI values of less than 1 in TF1-hPRL3 cells. In contrast, all the experimental points except at 0.2 fraction effect had CI values of more than 1 in TF1-pEGFP cells (Fig. 3c). The Fa-CI plot curve of the combination was below 1 for the effect range, indicating that the co-treatment of VS-5584 and ICG-001 was highly synergistic in TF1-hPRL3 cells. Conversely, most of CI values of greater than 1 implied that the co-administration of VS-5584 and ICG-001 was additive to antagonistic in TF1-pEGFP cells. We next determined whether this differential interaction between VS-5584 and ICG-001 is unique to this TF1 pair cells, we treated 2 additional AML cell lines with one PRL-3 high (MOLM-14) and one PRL-3 low (OCI-AML2), with VS-5584 and ICG-001, alone and in combination, monitored cell proliferation by CTG assay, and similarly analyzed the data for synergy. The expression of PRL-3 was 15-fold higher in MOLM-14 than in OCI-AML2 cells as quantified by qRT-PCR analysis (Additional file 2: Figure S1). Figure 3d showed that the resulting Fa-CI plot curve of VS-5844 and ICG-001 fell below 1 in MOLM-14 cells, but rise above 1 in OCI-AML2 cells.

The combination effect of VS-5844 and ICG-001 on inhibition of cell survival was also examined in primary AML sample with high or low PRL-3 expression. These primary AML cells also exhibited dose-dependent response to both drugs. With that, we calculated IC_50_ of these two drugs, and used the five-point constant ratio combination based on their IC_50_ values. A significantly lower cell viability percentage was recorded in samples subjected to combined treatment as compared to samples treated with single agent VS-5844 or ICG-001 in primary AML cells with high PRL-3, but not in samples with low PRL-3. The calculated CI values ranging from 0.29 to 0.68 also confirmed the synergism of this combination therapy in samples with high PRL-3. In contrast, the calculated CI values indicated an additive to antagonistic effect of this combination in patient samples with low PRL-3 (Table 1). The average of CI values of samples with high PRL-3 was statistically different from the average of CI values of samples with low PRL-3 (CI 0.42 vs 1.2, *p* = 0.008).

**Table 1:** Clinical characteristic of 10 AML patients and their CI values

Patient ID	Sex	Age (years)	FAB	Karyotype	FLT3	NPM1	PRL-3 status*	Average CI values
Pt#1	M	44	M5	47,XY,+8	FLT3-ITD	WT	35	0.29
Pt#2	M	70	M4	Normal	WT	N.A.	21	0.35
Pt#3	F	34	M4	Inv(16)(p13.1q22)	N.A.	N.A.	23	0.33
Pt#4	M	62	M3	add(14)(q24)	FLT3-ITD	Mutant	19	0.47
Pt#5	F	41	M2	Normal	FLT3-ITD	Mutant	15	0.68
Pt#6	F	37	M5	Normal	WT	N.A.	0.8	1.31
Pt#7	M	33	M4	t(3;7)(p25;q22)	N.A.	N.A.	1.9	0.96
Pt#8	F	75	M2	Normal	WT	N.A.	1.3	1.20
Pt#9	M	26	M0	Complex	WT	WT	0.7	1.45
Pt#10	F	68	M2	Normal	WT	Mutant	1.8	0.98

*Pt#* indicates unique patient number

*FAB* French-American-British, *Normal* normal karyotype, *FLT3* FMS-like tyrosine kinase 3, *ITD* internal tandem duplication, *NPM1* nucleophosmin 1, *N.A.* not available, *WT* wild type, *CI* combination index

*qRT-PCR analysis was applied to determine the expression of PRL-3 gene in a serial of primary AML samples. The baseline expression of PRL-3 in OCI-AML2 was used to normalize the fold changes of PRL-3 gene in primary AML cells. Here, patients with PRL-3 expression more than 10fold higher than its expression in OCI-AML2 cells were defined as PRL-3 high, while for patients whose PRL-3 expression were less than twofold higher or lower than its expression in OCI-AML2 cells, they were classified as PRL-3 low

Taken together, these results confirmed that the synergism of VS-5584 and ICG-001 was associated with the level of PRL-3 expression. Co-targeting PI3K/AKT and WNT/β-catenin pathways could achieve synergistic effects on AML cells overexpressing PRL-3.

### Enhanced cell death of AML cells by combination of VS-5584 and ICG-001

**Fig. 4:**
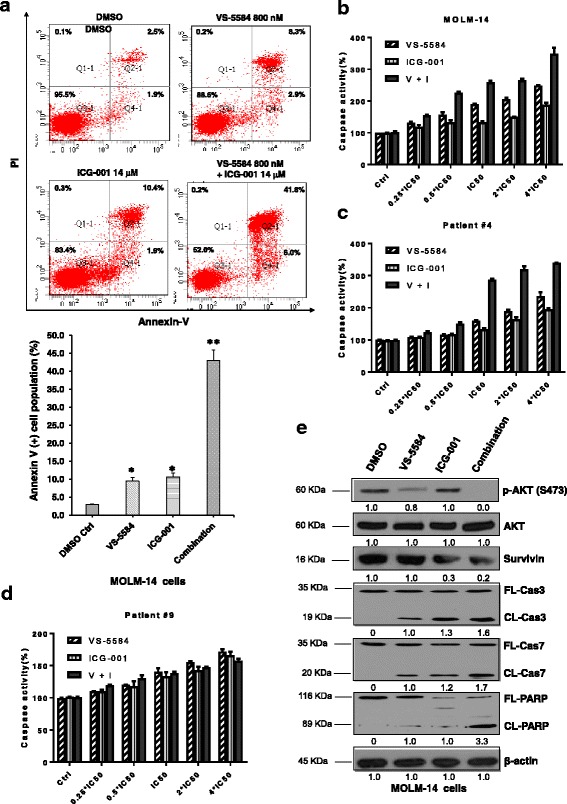
Evaluations of apoptotic response induced by simultaneous inhibition of AKT/mTOR and WNT/β-catenin pathways. **a** MOLM-14 cells were treated either with DMSO control, VS-5584, ICG-001 single agent or in combination for 48 h, harvested for staining with annexin V/FITC and PI dye as described in the “Methods” section. Upper representative images show the original FACS plots and lower bar figures represent the percentage of annexin V-positive cells, including both early and late apoptotic cells. **b** Luminescent assays for caspase 3 and caspase 7 activities in MOLM-14 cells and primary AML cells from unique patient number 4 (Pt#4, PRL-3 high) (**c**) incubated with either VS-5584, ICG-001 alone, or combination (*n* = 3, mean ± SD). Five-point constant ratio of combination-based single drug IC_50_ were used here. V + I: combination of VS-5584 and ICG-001. **d** Caspase 3 and caspase 7 activities in primary AML cells from unique patient number 9 (Pt#9, PRL-3 low) incubated with either VS-5584, ICG-001 alone, or combination (*n* = 3, mean ± SD). Five-point constant ratio of combination-based single drug IC_50_ were used here. V + I: combination of VS-5584 and ICG-001. **e** The cell lysates extracted from MOLM-14 cells treated with DMSO control, VS-5584 (800 nM), ICG-001 (14 μM) single agent or in combination for 48 h were subjected to Western blot analysis for AKT, p-AKT, Survivin, full length (FL) or cleaved (CL) caspase 3, 7 and PARP. Beta-actin was used as the loading control. Protein levels were determined by densitometric analysis. The experiments were duplicated, and representative images were shown

We evaluated whether this combined modality could stimulate the synergism of induction of apoptosis of PRL-3-high AML cells. To this end, MOLM-14 cells were incubated with DMSO, VS-5584 alone, ICG-001 alone, or combination of these two drugs for 48 h, followed by FACS analysis of apoptotic population. Single agent VS-5584 or ICG-001 treatment induced about 10% of cell death; however, combination of these two drugs together induced more than 40% of cell death (Fig. 4a). Two-way ANOVA analysis demonstrated there was a significant difference between the co-treatment and either of VS-5584 or of ICG-001 alone (*p* < 0.01).

The combination therapy is associated with increased activity of caspase 3/7 in MOLM-14 with high endogenous PRL-3. A strong activation of caspase 3/7 around 350% was observed in MOLM-14 cells when ICG-001 was used in combination with VS-5584, while the single-treated samples only showed caspase 3/7 activity of 114–250% (Fig. 4b, *p* < 0.01). We also performed caspase 3/7 assay in primary AML cells with high PRL-3 exposed to the combination therapy. Monotherapy with either VS-5584 or ICG-001 has moderate effect on caspase 3/7 activity in AML cells as the caspase activity increased around 30% at the presence of low doses of VS-5584 or ICG-001 tested (Fig. 4c). However, when ICG-001 was combined with VS-5584, the caspase 3/7 activity became much higher, and the significance of this increase was confirmed by two-way ANOVA (Fig. 4c, *p* < 0.01). Next, we examined the effect of combination of VS-5584 and ICG-001 on AML patient samples (patient no. 9) with low PRL-3 by using constant ratio combination therapy. As shown in Fig. 4d, ICG-001 or VS-5584 single treatment could increase caspase 3/7 activity to 160–170%, respectively. However, combination of these two drugs did not incite significant higher percentage of caspase 3/7 activity than treatment with single agent (Fig. 4d, *p* = 0.14) on PRL-3 low AML cells (patient no. 9). Overall, the combination treatment has significantly increased apoptotic cell population, enhanced activation of caspase 3/7, and magnified cleaved PARP preferentially in PRL-3 high AML cells.

Consistent with the results from FACS analysis and caspase 3/7 assays, western blot analysis showed that VS-5584 or ICG-001 monotherapy could lead to perceptible change of caspase 3, cleaved caspase 3, caspase 7, cleaved caspase 7, PARP, and cleaved PARP. Notably, western blot analysis indicated that cleavages of caspase 3, 7, and PARP were remarkably increased in MOLM-14 cells after incubation with VS-5584 and ICG-001 for 48 h (Fig. 4e). In addition, the target specificities of these inhibitors were confirmed by the downregulation of p-AKT and Survivin, a WNT target gene, respectively (Fig. 4e). However, when the same treatment regime was applied to OCI-AML2, a low PRL-3 cell line, we did not observe enhanced cleavage of caspase 3, 7, and PARP (Additional file 2: Figure S2). Collectively, combination of VS-5584 and ICG-001 selectively potentiates cell death of AML cells with high PRL-3.

In conclusion, VS-5584 significantly potentiates the proapoptotic effect of ICG-001. VS-5584 and ICG-001 combination has led to synergistic cell death of AML cell lines and primary AML cells. This synergism in killing is associated with the PRL-3 expression level. These results suggest that VS-5584 in combination with ICG-001 is a better therapy and may be beneficial for AML patients with high PRL-3.

### The combination of VS-5584 and ICG-001 causes significant survival benefit in AML xenograft mice

**Fig. 5:**
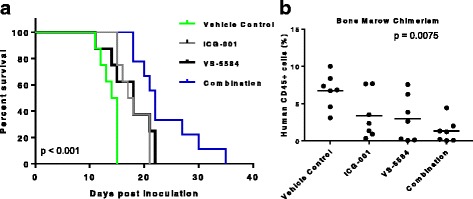
Co-treatment with VS-5584 and ICG-001 showed superior anti-AML efficacy in vivo in mouse xenograft models. **a** Mice were treated with vehicle control, VS-5584 5 mg/kg/day, ICG-001 50 mg/kg/day, or combination of two drugs respectively. Survival analysis showed that co-treatment with VS-5584 and ICG-001 significantly prolonged mice life than either of single treatment (*p* < 0.001). **b** Analysis of human CD45-positive cells in bone marrow samples harvested from mice at the endpoint of survival analysis by FACS method. Average of bone marrow chimerism was calculated as the average of 7 mice in each group ± SD (two-way ANOVA *p* = 0.0075)

We next determined whether this combination is also more efficacious against AML in in vivo settings. For this purpose, we used an AML xenograft mouse model. In this model, human MOLM-14 cells were injected into NOD/SCID mice through tail vein injection and treatments started 1 week after injection. The Kaplan-Meier survival curves from mice treated either with vehicle, each drug alone, or in combination were depicted in Fig. 5a. The median survival time from the vehicle-treated mice is 14.5 days, while the median survival time from ICG-001 alone treated or VS-5584 alone treated is 17.5 and 18 days, respectively, whereas the mice treated with the combination (median survival time 22 days) had significant longer survival time than either of single agent-treated mice (*p* < 0.01). The percentage of human CD45-positive cells in the mouse bone marrow determined by FACS analysis at the end of experiments. Bone marrow chimerism in mice treated with the VS-5584/ICG-001 combination was decreased to 1.3%. This is about 80% reduction as compared to vehicle-treated mice. The chimerism in mice treated with either ICG-001 or VS-5584 as single agents had an average percent reduction of 49% for ICG-001 and 56% for VS-5584 (Fig. 5b). There was a significant difference of chimerism change observed among treatment groups with statistical significance (*p* = 0.0075). Thus, the combination treatment of VS-5584 and ICG-001 is significantly more effective than single agent treatment and causes better survival and greater reduction in bone marrow chimerism in vivo. Moreover, the effect of the ICG-001/VS-5584 combination on leukemic burden and survival was also tested in OCI-AML2 (a PRL-3 low AML cell line) transplanted mice in the same way as for MOLM-14 xenograft mice. We found that the mice treated with ICG-001 and VS-5584 did not achieve a superior survival (median 18.5 days vs 19 days of ICG-001 group and 19.5 days of VS-5584 group, *p* > 0.05) than either of single drug alone (Additional file 2: Figure S3). FACS analysis of human CD45 showed that the OCI-AML2 BM burden in mice treated with combination therapy was not significantly different in single drug groups (*p* > 0.89, Additional file 2: Figure S3). Thus, taken together, in concordance with the in vitro data, ICG-001 and VS-5584 synergize to reduce leukemic burden and prolong survival selectively in PRL-3 high AML xenografts in mice and warrant further clinical investigation.

## Discussion

The unlimited proliferation and evasion of apoptosis as well as differentiation arrested are characteristic of AML cells [33, 34]. Deregulated signal transduction pathways, resulting from multiple genetic or epigenetic lesions, are believed to be the fundamental causes of the leukemogenesis [35–37]. Elevated expression of PRL-3 has been identified in about half of AML bone marrow samples and associated with poor outcome. To develop effective treatment, exploiting the vulnerability of AML cells and inhibition of these aberrant signal transduction pathways addicted by AML cells are necessary. In the current study, we employed a whole-genome shRNA library screening platform to identify vulnerabilities in PRL-3-high AML cells. Due to the fact that only portion of DNA extracted from each pool was used for PCR and subsequent sequencing, this may cause potential problems of inadequate representation of the shRNA pool in our screening method. We found and validated that AML cells expressing high PRL-3 are dependent on the AKT/mTOR and the WNT/β-catenin signaling pathways for survival.

PRL-3 is an oncogenic phosphatase with dual specificity, which can remove the phosphate group from both phosphotyrosine and phosphoserine/phosphothreonine residues within its target substrates [38]. A larger body of studies have revealed that, on average, PRL-3 is overexpressed in 22% of all types of solid tumors examined and in general, its overexpression is associated with cancer metastasis, advanced stage, and poor prognosis [39–42]. Increasing evidence from our group as well as other investigators have implicated a role for PRL3 in different hematological malignancies, including multiple myeloma (MM), AML, chronic myeloid leukemia (CML), and acute lymphoblastic leukemia (ALL) [15–17, 43–45]. Constitutive activation of AKT signaling pathways is one of the key steps in tumorigenesis-mediated by PRL-3 through downregulated PTEN [28]. As PI3K/AKT is an upstream regulator of the rapamycin-sensitive mTOR complex, mTOR signaling activity is accentuated in PRL-3-positive cancer cells [46]. Alternatively, an AKT-independent routine exists for the activation of mTOR complex where PRL-3 enhances mTOR binding affinity to Rag GTPases, leading to increased mTOR translocation to lysosomes [47].

On the other hand, we previously performed SILAC-based proteomic study and discovered that PRL-3 increased Leo-1 expression in AML [23]. Leo-1, a member of PAF complex, directly associates with β-catenin and activates WNT/β-catenin target genes [48]. Thus, PI3K/AKT and WNT/β-catenin pathways appear to be critical for the viability and maintenance of PRL-3 high AML. The activation of both pathways confers the aggressive phenotype of PRL-3 high AML. But at the same time, this high dependence of these two pathways for survival are also Achilles’ heel for PRL-3 high AML cells, which provides insights of the molecular mechanism underlying the synthetic lethality preferential in PRL-3 high AML cells over PRL-3 low AML cells. Here, we demonstrate that pharmacological intervention of these two pathways simultaneous by small molecular inhibitors results in profound synergism in PRL-3 high AML cells compared with PRL-3 low AML cells. Furthermore, this combinatorial therapy with VS-5584 and ICG-001 validates this novel synthetic lethality principle in in vivo models and in primary AML patient samples with high PRL-3. It is notable to point out that this combination therapy may be effective in other type of cancers expressing high PRL-3 protein, but this will need to be verified.

## Conclusions

Taken together, our work uncovers a novel synthetic lethality through combinatory therapy of co-targeting PI3K/AKT and WNT/β-catenin pathways as a valuable druggable targets for PRL-3 high AML and provides strong rationale to further develop these strategies to treat this subtype of AML patients with high PRL-3 who are associated with poor prognosis in clinic.

## Additional files

Additional file 1: **Table S1.** Genes critical for survival of PRL-3 high AML cells revealed by Mission shRNA library screening. (XLS 246 kb)

Additional file 2: **Figure S1.** Relative quantification of PTP4A3 (PRL-3) expression level in OCI-AML2 and MOLM-14 cells by qRT-PCR analysis. **Figure S2.** Representative immunoblot showing the levels of indicated proteins in OCI-AML2 (PRL-3 low) cells with different treatments. **Figure S3.** In vivo efficacy of VS-5584, ICG-001 single agent and combination treatment in mouse xenograft models transplanted OCI-AML2 cells. (PDF 246 kb)

## Abbreviations

AML: Acute myeloid leukemia
CI: Combination index
DMSO: Dimethyl sulfoxide
mTOR: The mammalian target of rapamycin
PI3K: Phosphatidylinositol-3-kinase
PRL-3: Phosphatase of regenerating liver 3
PTP: Protein tyrosine phosphatase

## References

[1] Zhou J, Chng WJ (2014). Identification and targeting leukemia stem cells: the path to the cure for acute myeloid leukemia. World J Stem Cells.

[2] Hackl H, Astanina K, Wieser R (2017). Molecular and genetic alterations associated with therapy resistance and relapse of acute myeloid leukemia. J Hematol Oncol.

[3] Saygin C, Carraway HE (2017). Emerging therapies for acute myeloid leukemia. J Hematol Oncol.

[4] Zhu X, Ma Y, Liu D (2010). Novel agents and regimens for acute myeloid leukemia: 2009 ASH annual meeting highlights. J Hematol Oncol.

[5] Khaled S, Al Malki M, Marcucci G (2016). Acute myeloid leukemia: biologic, prognostic, and therapeutic insights. Oncology (Williston Park).

[6] Zhou J, Goh BC, Albert DH, Chen CS (2009). ABT-869, a promising multi-targeted tyrosine kinase inhibitor: from bench to bedside. J Hematol Oncol.

[7] Bessette DC, Qiu D, Pallen CJ (2008). PRL PTPs: mediators and markers of cancer progression. Cancer Metastasis Rev.

[8] Rubio T, Kohn M (2016). Regulatory mechanisms of phosphatase of regenerating liver (PRL)-3. Biochem Soc Trans.

[9] Stephens BJ, Han H, Gokhale V, Von Hoff DD (2005). PRL phosphatases as potential molecular targets in cancer. Mol Cancer Ther.

[10] Fagerli UM, Holt RU, Holien T, Vaatsveen TK, Zhan F, Egeberg KW, Barlogie B, Waage A, Aarset H, Dai HY (2008). Overexpression and involvement in migration by the metastasis-associated phosphatase PRL-3 in human myeloma cells. Blood.

[11] Abdollahi P, Vandsemb EN, Hjort MA, Misund K, Holien T, Sponaas AM, Ro TB, Slordahl TS, Borset M (2017). Src family kinases are regulated in multiple myeloma cells by phosphatase of regenerating Liver-3. Mol Cancer Res.

[12] Campbell AM, Zhang ZY (2014). Phosphatase of regenerating liver: a novel target for cancer therapy. Expert Opin Ther Targets.

[13] Cramer JM, Zimmerman MW, Thompson T, Homanics GE, Lazo JS, Lagasse E (2014). Deletion of Ptp4a3 reduces clonogenicity and tumor-initiation ability of colitis-associated cancer cells in mice. Stem Cell Res.

[14] Zhou J, Bi C, Chng WJ, Cheong LL, Liu SC, Mahara S, Tay KG, Zeng Q, Li J, Guo K (2011). PRL-3, a metastasis associated tyrosine phosphatase, is involved in FLT3-ITD signaling and implicated in anti-AML therapy. PLoS One.

[15] Beekman R, Valkhof M, Erkeland SJ, Taskesen E, Rockova V, Peeters JK, Valk PJ, Lowenberg B, Touw IP (2011). Retroviral integration mutagenesis in mice and comparative analysis in human AML identify reduced PTP4A3 expression as a prognostic indicator. PLoS One.

[16] Park JE, Yuen HF, Zhou JB, Al-Aidaroos AQ, Guo K, Valk PJ, Zhang SD, Chng WJ, Hong CW, Mills K, Zeng Q (2013). Oncogenic roles of PRL-3 in FLT3-ITD induced acute myeloid leukaemia. EMBO Mol Med.

[17] Qu S, Liu B, Guo X, Shi H, Zhou M, Li L, Yang S, Tong X, Wang H (2014). Independent oncogenic and therapeutic significance of phosphatase PRL-3 in FLT3-ITD-negative acute myeloid leukemia. Cancer.

[18] Zhou J, Chan ZL, Bi C, Lu X, Chong PS, Chooi JY, Cheong LL, Liu SC, Ching YQ, Zhou Y (2017). LIN28B activation by PRL-3 promotes leukemogenesis and a stem cell-like transcriptional program in AML. Mol Cancer Res.

[19] Sharlow ER, Wipf P, McQueeney KE, Bakan A, Lazo JS (2014). Investigational inhibitors of PTP4A3 phosphatase as antineoplastic agents. Expert Opin Investig Drugs.

[20] Kim KA, Song JS, Jee J, Sheen MR, Lee C, Lee TG, Ro S, Cho JM, Lee W, Yamazaki T (2004). Structure of human PRL-3, the phosphatase associated with cancer metastasis. FEBS Lett.

[21] Kozlov G, Cheng J, Ziomek E, Banville D, Gehring K, Ekiel I (2004). Structural insights into molecular function of the metastasis-associated phosphatase PRL-3. J Biol Chem.

[22] Zeng Q, Si X, Horstmann H, Xu Y, Hong W, Pallen CJ (2000). Prenylation-dependent association of protein-tyrosine phosphatases PRL-1, -2, and -3 with the plasma membrane and the early endosome. J Biol Chem.

[23] Chong PS, Zhou J, Cheong LL, Liu SC, Qian J, Guo T, Sze SK, Zeng Q, Chng WJ (2014). LEO1 is regulated by PRL-3 and mediates its oncogenic properties in acute myelogenous leukemia. Cancer Res.

[24] Zhou J, Pan M, Xie Z, Loh SL, Bi C, Tai YC, Lilly M, Lim YP, Han JH, Glaser KB (2008). Synergistic antileukemic effects between ABT-869 and chemotherapy involve downregulation of cell cycle-regulated genes and c-Mos-mediated MAPK pathway. Leukemia.

[25] Zhou J, Bi C, Janakakumara JV, Liu SC, Chng WJ, Tay KG, Poon LF, Xie Z, Palaniyandi S, Yu H (2009). Enhanced activation of STAT pathways and overexpression of survivin confer resistance to FLT3 inhibitors and could be therapeutic targets in AML. Blood.

[26] Jiang Y, Liu XQ, Rajput A, Geng L, Ongchin M, Zeng Q, Taylor GS, Wang J (2011). Phosphatase PRL-3 is a direct regulatory target of TGFbeta in colon cancer metastasis. Cancer Res.

[27] Stephens B, Han H, Hostetter G, Demeure MJ, Von Hoff DD (2008). Small interfering RNA-mediated knockdown of PRL phosphatases results in altered Akt phosphorylation and reduced clonogenicity of pancreatic cancer cells. Mol Cancer Ther.

[28] Wang H, Quah SY, Dong JM, Manser E, Tang JP, Zeng Q (2007). PRL-3 down-regulates PTEN expression and signals through PI3K to promote epithelial-mesenchymal transition. Cancer Res.

[29] Hu S, Ueda M, Stetson L, Ignatz-Hoover J, Moreton S, Chakrabarti A, Xia Z, Karan G, de Lima M, Agrawal MK, Wald DN (2016). A novel glycogen synthase kinase-3 inhibitor optimized for acute myeloid leukemia differentiation activity. Mol Cancer Ther.

[30] McCubrey JA, Steelman LS, Bertrand FE, Davis NM, Abrams SL, Montalto G, D'Assoro AB, Libra M, Nicoletti F, Maestro R (2014). Multifaceted roles of GSK-3 and Wnt/beta-catenin in hematopoiesis and leukemogenesis: opportunities for therapeutic intervention. Leukemia.

[31] Kolev VN, Wright QG, Vidal CM, Ring JE, Shapiro IM, Ricono J, Weaver DT, Padval MV, Pachter JA, Xu Q (2015). PI3K/mTOR dual inhibitor VS-5584 preferentially targets cancer stem cells. Cancer Res.

[32] Zhao Y, Masiello D, McMillian M, Nguyen C, Wu Y, Melendez E, Smbatyan G, Kida A, He Y, Teo JL, Kahn M (2016). CBP/catenin antagonist safely eliminates drug-resistant leukemia-initiating cells. Oncogene.

[33] Zhou J, Lu X, Tan TZ, Chng WJ (2018). X-linked inhibitor of apoptosis inhibition sensitizes acute myeloid leukemia cell response to TRAIL and chemotherapy through potentiated induction of proapoptotic machinery. Mol Oncol.

[34] Nowak D, Stewart D, Koeffler HP (2009). Differentiation therapy of leukemia: 3 decades of development. Blood.

[35] Sakamoto KM, Grant S, Saleiro D, Crispino JD, Hijiya N, Giles F, Platanias L, Eklund EA (2015). Targeting novel signaling pathways for resistant acute myeloid leukemia. Mol Genet Metab.

[36] Zhou J, Ching YQ, Chng WJ (2015). Aberrant nuclear factor-kappa B activity in acute myeloid leukemia: from molecular pathogenesis to therapeutic target. Oncotarget.

[37] Zhou J, Chng WJ (2017). Aberrant RNA splicing and mutations in spliceosome complex in acute myeloid leukemia. Stem Cell Investig.

[38] Al-Aidaroos AQ, Zeng Q (2010). PRL-3 phosphatase and cancer metastasis. J Cell Biochem.

[39] Basak S, Jacobs SB, Krieg AJ, Pathak N, Zeng Q, Kaldis P, Giaccia AJ, Attardi LD (2008). The metastasis-associated gene Prl-3 is a p53 target involved in cell-cycle regulation. Mol Cell.

[40] Lian S, Meng L, Yang Y, Ma T, Xing X, Feng Q, Song Q, Liu C, Tian Z, Qu L, Shou C (2017). PRL-3 promotes telomere deprotection and chromosomal instability. Nucleic Acids Res.

[41] Wang H, Vardy LA, Tan CP, Loo JM, Guo K, Li J, Lim SG, Zhou J, Chng WJ, Ng SB (2010). PCBP1 suppresses the translation of metastasis-associated PRL-3 phosphatase. Cancer Cell.

[42] den Hollander P, Rawls K, Tsimelzon A, Shepherd J, Mazumdar A, Hill J, Fuqua SA, Chang JC, Osborne CK, Hilsenbeck SG (2016). Phosphatase PTP4A3 promotes triple-negative breast cancer growth and predicts poor patient survival. Cancer Res.

[43] Broyl A, Hose D, Lokhorst H, de Knegt Y, Peeters J, Jauch A, Bertsch U, Buijs A, Stevens-Kroef M, Beverloo HB (2010). Gene expression profiling for molecular classification of multiple myeloma in newly diagnosed patients. Blood.

[44] Gronroos T, Teppo S, Mehtonen J, Laukkanen S, Liuksiala T, Nykter M, Heinaniemi M, Lohi O (2017). Overexpression of PTP4A3 in ETV6-RUNX1 acute lymphoblastic leukemia. Leuk Res.

[45] Zhou J, Cheong LL, Liu SC, Chong PS, Mahara S, Bi C, Ong KO, Zeng Q, Chng WJ (2012). The pro-metastasis tyrosine phosphatase, PRL-3 (PTP4A3), is a novel mediator of oncogenic function of BCR-ABL in human chronic myeloid leukemia. Mol Cancer.

[46] Dienstmann R, Rodon J, Serra V, Tabernero J (2014). Picking the point of inhibition: a comparative review of PI3K/AKT/mTOR pathway inhibitors. Mol Cancer Ther.

[47] Ye Z, Al-Aidaroos AQ, Park JE, Yuen HF, Zhang SD, Gupta A, Lin Y, Shen HM, Zeng Q (2015). PRL-3 activates mTORC1 in cancer progression. Sci Rep.

[48] Muntean AG, Tan J, Sitwala K, Huang Y, Bronstein J, Connelly JA, Basrur V, Elenitoba-Johnson KS, Hess JL (2010). The PAF complex synergizes with MLL fusion proteins at HOX loci to promote leukemogenesis. Cancer Cell.

