# Supplementary material for: A loss-of-function genetic screening reveals synergistic targeting of AKT/mTOR and WTN/β-catenin pathways for treatment of AML with high PRL-3 phosphatase
Source: J Hematol Oncol. 2018 Mar 7;11:36. doi: 10.1186/s13045-018-0581-9 (PMC5842526; doi:10.1186/s13045-018-0581-9)
Supplement: Supplementary file 2 — Figure S1. Relative quantification of PTP4A3 (PRL-3) expression level in OCI-AML2 and MOLM-14 cells by qRT-PCR analysis. Figure S2. Representative immunoblot showing the levels of indicated proteins in OCI-AML2 (PRL-3 low) cells with different treatments. Figure S3. In vivo efficacy of VS-5584, ICG-001 single agent and combination treatment in mouse xenograft models transplanted OCI-AML2 cells. (PDF 246 kb) [file 13045_2018_581_MOESM2_ESM.pdf]

Supplementary Figures

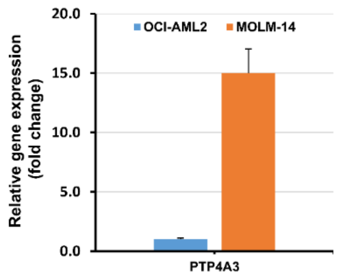

Supplementary Figure S1. Relative quantification of PTP4A3 (PRL-3) expression level in OCI-AML2 and MOLM-14 cells by qRT-PCR analysis. The expression of PRL-3 gene in MOLM-14 cells was 15 times higher than its expression in OCI-AML3 cells after normalization with their GAPDH level, respectively (n = 3, mean ± SD). The average CT value of PRL-3 was 23.67 in MOLM-14 cells and 28.07 in OCI-AML2 cells.

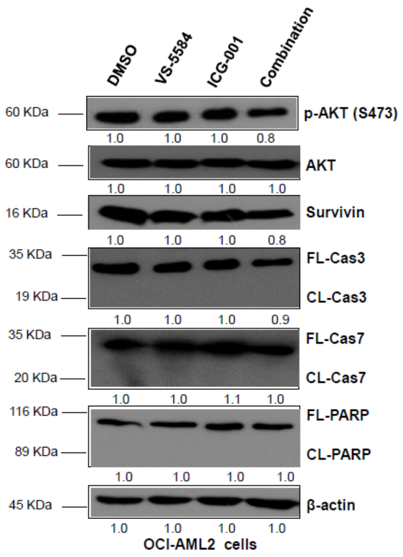

Supplementary Figure S2. Representative immunoblot showing the levels of indicated proteins in OCI-AML2 (PRL-3 low) cells with different treatments. The cell lysates extracted from OCI-AML2 cells treated with DMSO control, VS-5584 (800 nM), ICG-001 (14 μM) single agent or in combination for 48 hours were subjected to Western blot analysis for AKT, p-AKT, Survivin, full length (FL) or cleaved (CL) caspase 3, 7 and PARP. Beta-actin was used as loading

control. Protein levels were determined by densitometric analysis. The experiments were duplicated and representative images were shown.

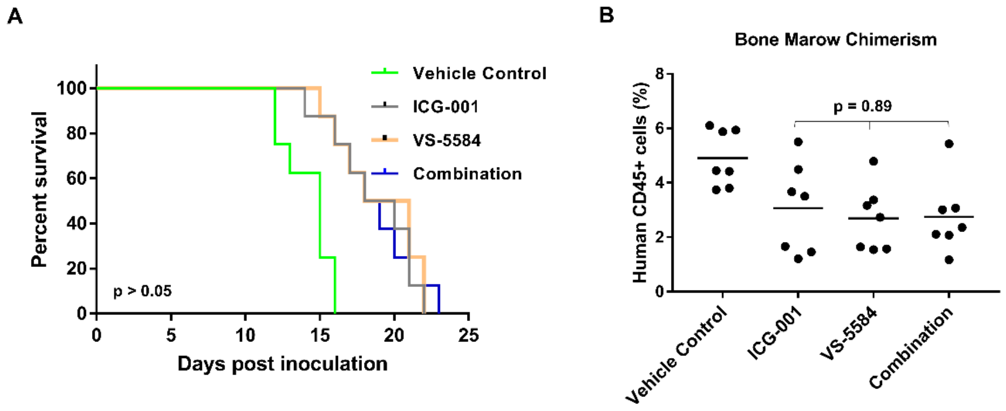

Supplementary Figure S3. *In vivo* efficacy of VS-5584, ICG-001 single agent and combination treatment in mouse xenograft models transplanted OCI-AML2 cells. (A) Mice were treated with vehicle control, VS-5584 5 mg/kg/day, ICG-001 50 mg/kg/day or combination of two drugs respectively. Survival analysis showed that co-treatment with VS-5584 and ICG-001 didn't produce additional benefit in survival when compared to either of single treatment ($p < 0.05$). **(B)** Analysis of human CD45 positive cells in bone marrow samples harvested from xenograft mice at the endpoint of survival analysis by FACS method. Average of bone marrow chimerism was calculated as the average of 7 mice in each group ± SD (two-way ANOVA $p = 0.89$).
